# Nanotechnology‐facilitated vaccine development during the coronavirus disease 2019 (COVID‐19) pandemic

**DOI:** 10.1002/EXP.20210082

**Published:** 2022-07-21

**Authors:** Ziqi Wang, Kai Cui, Ulrich Costabel, Xiaoju Zhang

**Affiliations:** ^1^ Department of Respiratory and Critical Care Medicine Zhengzhou University People's Hospital Henan Provincial People's Hospital Zhengzhou Henan P. R. China; ^2^ Academy of Medical Science Zhengzhou University Zhengzhou Henan P. R. China; ^3^ Department of Pneumology Ruhrlandklinik University Medicine Essen Essen Germany

**Keywords:** COVID‐19 vaccine, nanotechnology, SARS‐CoV‐2

## Abstract

Coronavirus disease 2019 (COVID‐19) continually poses a significant threat to the human race, and prophylactic vaccination is the most potent approach to end this pandemic. Nanotechnology is widely adopted during COVID‐19 vaccine development, and the engineering of nanostructured materials such as nanoparticles has opened new possibilities in innovative vaccine development by improving the design and accelerating the development process. This review aims to comprehensively understand the current situation and prospects of nanotechnology‐enabled vaccine development against the COVID‐19 pandemic, with an emphasis on the interplay between nanotechnology and the host immune system.

## Graphical abstract

This review comprehensively summarizes the advantages that nanotechnology holds for vaccine development, as well as the current situation of nanotechnology‐enabled vaccine development against the COVID‐19 pandemic, with a focus on the interplay between nanotechnology and the host immune system.

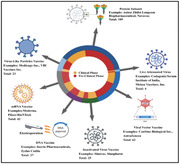

## 1 INTRODUCTION

Coronavirus disease 2019 (COVID‐19) continually poses a great threat to the human race, with a total of over 1.8 hundred million confirmed cases and approximately 4 million deaths reported by the World Health Organization (WHO) by August 2021, and is still raging globally.^[^ ^1^ ^]^ Severe acute respiratory syndrome coronavirus 2 (SARS‐CoV‐2) is the etiological factor of COVID‐19. It's a single‐stranded RNA (ssRNA) virus^[^ ^2^ ^]^ that has four structural proteins, the spike (S), envelope (E), membrane (M), and nucleolus side (N), all of which elicit an immune response.^[^ ^3^ ^]^ Evidence shows that SARS‐CoV‐2 utilizes the C‐terminal structural domain (CTD) of S1 subunit of the S protein as a receptor‐binding structural domain (RBD) to bind the target cell receptors, including angiotensin‐converting enzyme 2 (ACE2).^[^ ^4^ ^]^ Therefore, S and RBD are currently the most used target proteins for vaccines and drugs aiming at COVID‐19.

**FIGURE 1:**
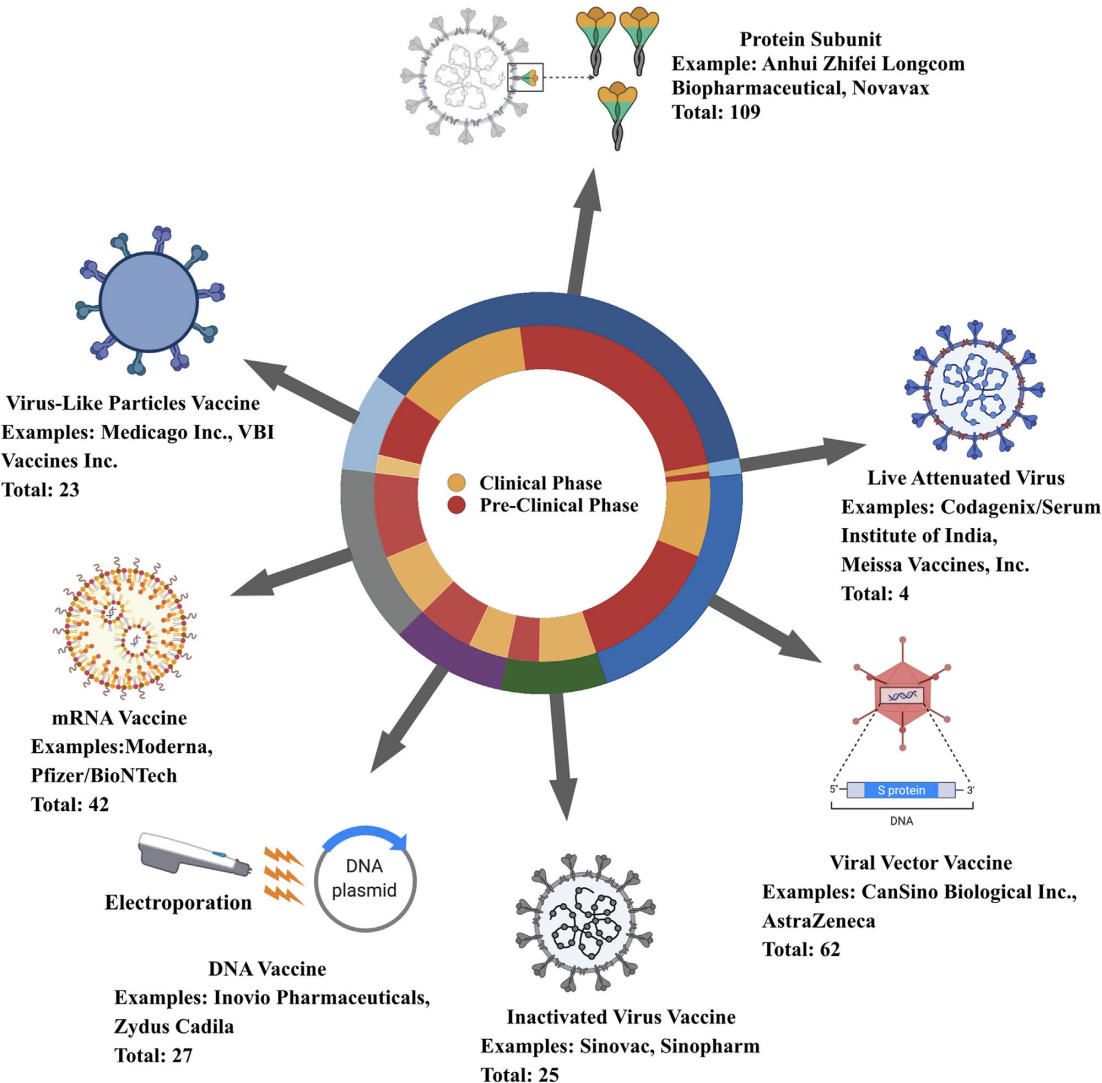
A glance at the coronavirus disease 19 (COVID‐19) vaccine landscape. Data are derived from the World Health Organization (WHO) database.^[^ ^6^ ^]^ Created in BioRender.com (https://biorender.com/)

**FIGURE 2:**
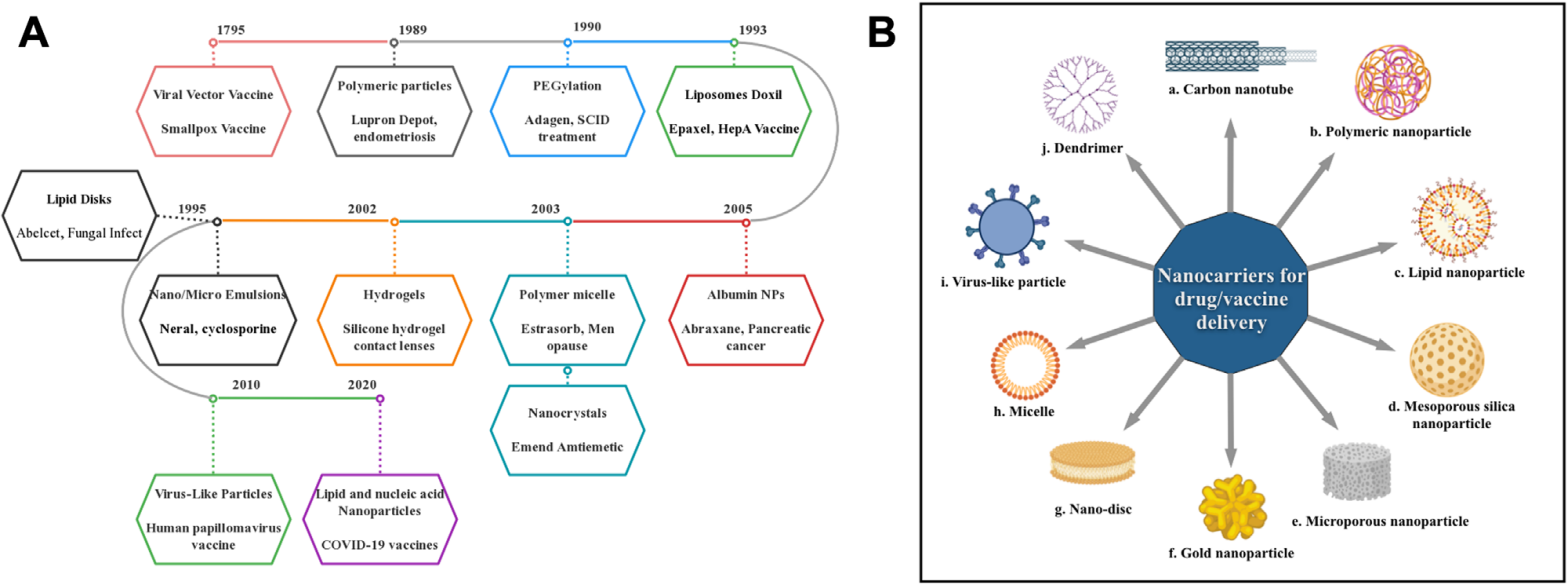
Timeline of nanotechnology and nanocarriers approved by FDA. (A) Timeline of the FDA approval of nanotechnology for therapeutic and vaccine applications.^[^ ^177^, ^178^ ^]^ Listed contents are not to scale with each other. (B) A summary of nanocarriers commonly used for drug and vaccine delivery: (a) Carbon nanotubes. They have a basic structure made of graphene, which is formed by carbon atoms densely organized in a regular sp2‐bonded atomic‐scale honeycomb (hexagonal) pattern. They can be classified into single and multi‐walled carbon nanotubes, functionalized with peptides, proteins, nucleic acids, and drugs and displayed low toxicity and immunogenic.^[^ ^14^ ^]^ (b) Polymeric nanoparticles generally including two distinct types, one is nanocapsules that have an oil or aqueous core with a polymeric shell surrounded, the solubility can be improved so the drug can dissolve in the inner core, and the shell are aiming to protect the drug from the environment and control the releases of the drug. Another type of polymeric nanoparticle is nanosphere, which is constructed based on polymeric network or combined with other material like metal.^[^ ^18^ ^]^ (c) Lipid nanoparticles are discussed in detail in the following parts of the review. (d) Mesoporous nanoparticle is a nanoscale porous material with a pore size between 2 and 50 nm, mostly made from silica. Mesoporous silica nanoparticle (MSNP) is one of the most well‐studied inorganic nanoparticles, with a unique mesoporous structure that allows for controlled drug delivery, with special emphasis in cancer treatment.^[^ ^16^ ^]^ (e) Microporous nanoparticle is nanoscale porous material with pa ore size smaller than 2 nm and can be made from carbon, polymer, and silica, among others.^[^ ^17^ ^]^ (f) Gold nanoparticles consist of a gold core, with sizes ranging from 1 to 150 nm and a surface coating that could be modified. The gold nanoparticles hold several advantages for drug delivery, including the ease of synthesis, the inertness, and non‐toxicity of the gold core, the ready functionalization, generally through thiol linkages, and various methods for triggering drug release at remote place.^[^ ^10^ ^]^ (g) Nanodisc or nanodisk generally refers to drug‐loaded discoidal reconstituted high‐density lipoprotein (rHDL) particles, which are composed of phospholipids bilayer and recombinant apolipoprotein “scaffold.”^[^ ^13^ ^]^ (h) Micelles are spherical amphiphilic structures formed from supramolecular assemble of amphiphilic molecules (surfactants), with a hydrophilic surface and a hydrophobic core that enables encapsulation of drugs.^[^ ^15^ ^]^ (i) Virus‐like particles are discussed in detail in the following parts of this review. (j) Dendrimers are highly organized, branched polymeric molecules, usually they have a monodispersing structure, consisting of a polymer inner core and symmetric branching units. The interest in dendrimers can be attributed to their high loading of drugs, water solubility, modifiable surface functionality et al.^[^ ^12^, ^104^ ^]^ (B) was created in BioRender.com (https://biorender.com/)

Vaccines are now considered the most potent approach to end this pandemic, which may decrease infection, transmission, intensive care unit admission, and death by generating long‐lasting immunity, ultimately resulting in disease control or eradication. Significant efforts have been made cooperatively to achieve this shared goal. The earliest came as soon as the nucleic sequence was made publicly accessible by the China Center for Disease Control and Prevention (China CDC). Thereafter, Moderna and BioNTech took the lead in pushing mRNA vaccine candidates into clinical trials at an unprecedented speed of less than 1 month and received clinical use authorization within 1 year, breaking the latest speed record of 4 years kept by the mumps vaccine.^[^ ^5^ ^]^ There are approximately 300 vaccines in development to date. They can generally be classified into six categories: live attenuated virus vaccine, inactivated virus vaccine, viral vector vaccine, protein subunit vaccine, RNA vaccine, and DNA vaccine, which are briefly summarized in Figure 1. Detailed information was obtained from the WHO database.^[^ ^6^ ^]^ Nanotechnology is broadly defined based on a particle size around 1,000 nm or blow, and with the multiple functions that are attributed to its dimension.^[^ ^7^, ^8^, ^9^ ^]^ Various nanomaterials have been widely adopted as nanocarriers for drug delivery (Figure 2).^[^ ^10^, ^11^, ^12^, ^13^, ^14^, ^15^, ^16^, ^17^, ^18^ ^]^ Meanwhile, nanotechnology has also greatly facilitated vaccine development, from antigen delivery to immune response boosting, allowing a nano‐level perspective that enables scientists to better mimic the natural interplay between viruses and the immune system.^[^ ^19^ ^]^ This review will provide an overview on the topic of COVID‐19 vaccine development, with an emphasis on the role of nanotechnology during this process.

## 2 BACKGROUND: A BRIEF INTRODUCTION OF VACCINE IMMUNOLOGY AND THE ADVANTAGES THAT NANOTECHNOLOGY HOLDS FOR VACCINE DEVELOPMENT

Improving the immunogenicity of the vaccine with an acceptable safety profile is the final goal, regardless of the technology applied; thus, it is important to understand vaccination immunology before understanding how nanotechnology is benefiting the vaccine design.

The human defense system can be classified into three categories: (1) physical and chemical barriers; (2) the innate immune system composed of dendritic cells (DCs), macrophages, granulocytes (neutrophils, eosinophils, and basophils), natural killer (NK) cells, and the complement system; (3) and the adaptive immune system consisting of T and B lymphocytes. The main role of the innate immune system in vaccination is to recognize, take up, process, and present vaccine antigens to T and B cells and assist in the activation of the adaptive immune system. Upon activation, the adaptive immune system neutralizes and eliminates pathogens through humoral and cellular immune responses.^[^ ^20^ ^]^ Furthermore, the adaptive immune system can generate an immune memory after effective activation, and once this memory is established, the adaptive immune system produces a faster and stronger immune response upon exposure to the same pathogen,^[^ ^21^ ^]^ leading to its efficient elimination. Establishing long‐term cellular and humoral immunological memories is the main purpose of vaccination.^[^ ^22^ ^]^

Upon injection of a vaccine into the human body, the innate immune system quickly elicits non‐specific immunity within minutes to hours in response to external antigens.^[^ ^23^ ^]^ This process is mediated by the interaction between the antigens or adjuvants mimicking the pathogen‐associated molecular patterns (PAMPs) with the corresponding pattern‐recognition receptors (PRRs) such as toll‐like receptors (TLRs) and nucleotide‐binding oligomerization domain‐like receptors, which are expressed on the surface of innate immune cells or on endosomes inside, especially DCs,^[^ ^24^ ^]^ which can sense different signals. The maturation of DCs is promoted by interactions between PAMPs and PRRs.^[^ ^25^ ^]^ While immature DCs are able to effectively uptake and process antigens, only mature DCs are capable of efficiently presenting antigens.^[^ ^26^ ^]^ Therefore, it is crucial that a vaccine effectively activates DCs. Efforts have been made to achieve this, including adding adjuvants to vaccines, for instance, CpG 1018, which can be detected by DCs through TLR9, and mRNAs through TLR3, TLR7, and TLR8.^[^ ^27^ ^]^ Nanoparticles can be used as vaccine carriers to protect antigens from biodegradation, maintain their native conformation, and enable the simultaneous delivery of antigens and adjuvants,^[^ ^28^ ^]^ resulting in more effective vaccine delivery. Additionally, some nanoparticles have the ability to create a depot effect at the injection site,^[^ ^29^, ^30^ ^]^ allowing slow release of antigens and prolonged exposure, thus boosting innate immunity. Additionally, antigens encapsulated in nanoparticles are preferentially taken up by DCs compared with soluble antigens.^[^ ^31^ ^]^ The size, surface charge, and superficial structure of nanoparticles can be optimized to improve this advantage;^[^ ^32^ ^]^ however, it is worth mentioning that over‐activation of the innate immune system may have the opposite effect, as shown in the development of mRNA vaccines, where excessive cytokine secretion induces DCs activation and elicits an intracellular antiviral response that interferes with the expression of external mRNA, resulting in inadequate antigen production.^[^ ^33^, ^34^ ^]^

After antigen uptake, DCs enter the drainage lymph nodes via either the high endothelial venules or lymph vessels.^[^ ^35^, ^36^ ^]^ Presentation of the processed antigen to naïve T cells by DCs depends on three distinct pathways, which are the classical major histocompatibility complex (MHC)‐I‐CD8+ T cells pathway, the classical MHC‐II‐CD4+ T cells pathway, and the cross‐presentation pathway,^[^ ^37^ ^]^ along with the secretion of cytokines such as type I interferons and interleukin‐6 (IL‐6) by matured DCs, which are also important for the activation of an adaptive system.^[^ ^25^ ^]^ Vaccine particles are usually taken up by DCs through endocytosis and endosome formation, followed by digestion in lysosomes of DCs, after which the generated peptides are loaded onto the MHC‐II molecules to form MHC‐II‐peptide complexes in specialized endosomal compartments before being expressed on the surface of antigen‐presenting cells (APCs). Finally, the peptides are present strictly in naïve CD4+ T cells.^[^ ^37^ ^]^ T‐cell receptors (TCRs) on the surface of naïve CD4+ T cells effectively bind to MHC‐II‐peptide complexes, finally leading to the proliferation and differentiation into different types of effector cells, depending on the cytokine milieu of the microenvironment^[^ ^38^ ^]^ that classically includes T‐helper 1 (Th1) and T‐helper 2 (Th2) cells, which are ready to provide the signal for B cell activation through the CD40 ligand. The lack of an effective cellular immune response mediated by CD8+ T cells is a major defect of traditional vaccines,^[^ ^39^ ^]^ given that they rarely activate the classical MHC‐I‐mediated presentation and cross‐presentation pathway. Nanotechnology could be useful in this scenario, given that nanoparticles can be designed to effectively activate the cross‐presentation pathway through the release of antigens into the cytoplasm after being packaged into the endosome, a process called endosome escape, which can be realized in a pH‐responsive or photic manner.^[^ ^40^ ^]^ Other mechanisms have also been reported.^[^ ^41^ ^]^

In addition to being taken up by migrated DCs at the injection site, vaccine particles can directly accumulate in draining lymph nodes, and then bind to B cell receptors (BCRs) on the surface of B cells in the germinal center (GC) by direct interaction or through the presentation by resident APCs, mainly follicular dendritic cells (FDCs) and macrophages in the subcapsular sinus and medulla of lymph nodes in an MHC‐independent manner.^[^ ^42^ ^]^ Antigen binding leads to BCR clustering and delivers the first signal for B cell activation, and activated Th cells provide a second signal for B cell activation through CD40/CD40 ligand interaction. Activated B cells then proliferate, differentiate, and somatically hypermutate to produce high‐affinity antibodies with the assistance of follicular helper T (Tfh) cells.^[^ ^43^ ^]^ Nanotechnology has also been widely adopted in vaccine development to improve this process and produce robust humoral immunity. There are generally three approaches for nanoparticles to achieve this: to increase the accumulation of antigens in the draining lymph site, to display the antigens in a repetitive and organized way to BCRs that mimic the natural structure of a virus, and to improve the cooperation of Tfh cells with GC B cells.^[^ ^44^ ^]^ Antigens conjugated to nanoparticles can significantly improve lymph node accumulation compared to free antigens, which is widely observed in multiple nanoparticle‐based vaccines.^[^ ^45^, ^46^ ^]^ It is well established that the size of vaccine particles is inversely proportional to the lymph node accumulation of antigens, generally, particles with a size between ∼20 and 200 nm could freely drain to the lymph nodes, and the ones with a size between ∼500 and 2,000 nm are prone to retain at the injection sites and are captured by migrated DCs.^[^ ^47^ ^]^ The modification of the nanoparticles may further improve the ability of lymph node targeting,^[^ ^48^ ^]^ for instance, incorporation of phosphatidylserine with liposomes significantly increased lymph node accumulation by three‐folds.^[^ ^47^ ^]^

Additionally, nanoparticles can be artificially modified so that the antigen displayed on their surface mimics the natural structure of the PAMPs, which is vital for optimizing the innate and humoral immune responses. Many innate immune system sensors, including TLRs, are multimeric (pentamer or decamer),^[^ ^49^ ^]^ leading to high avidity when binding to repetitive antigens. Research has also shown that nanoparticles such as virus‐like particles (VLPs) can utilize the complement system to improve the deposition of antigens on FDCs.^[^ ^50^ ^]^ Furthermore, repetitiveness facilitates B cell activation. Repetitive and organized antigens promote the cross‐linking and clustering of BCR,^[^ ^51^ ^]^ which is required for the intercellular signaling of B cell activation. Moreover, highly repetitive and organized antigens carry the features of T‐independent antigens such as haptenated polymers, which can activate B cells without the costimulatory signal provided by CD4+ T cells,^[^ ^52^ ^]^ thus reducing the dependency on Th cells for B cell activation. Together, these result in an enhanced uptake of antigens by APCs and a lower threshold of B cell activation.

**FIGURE 3:**
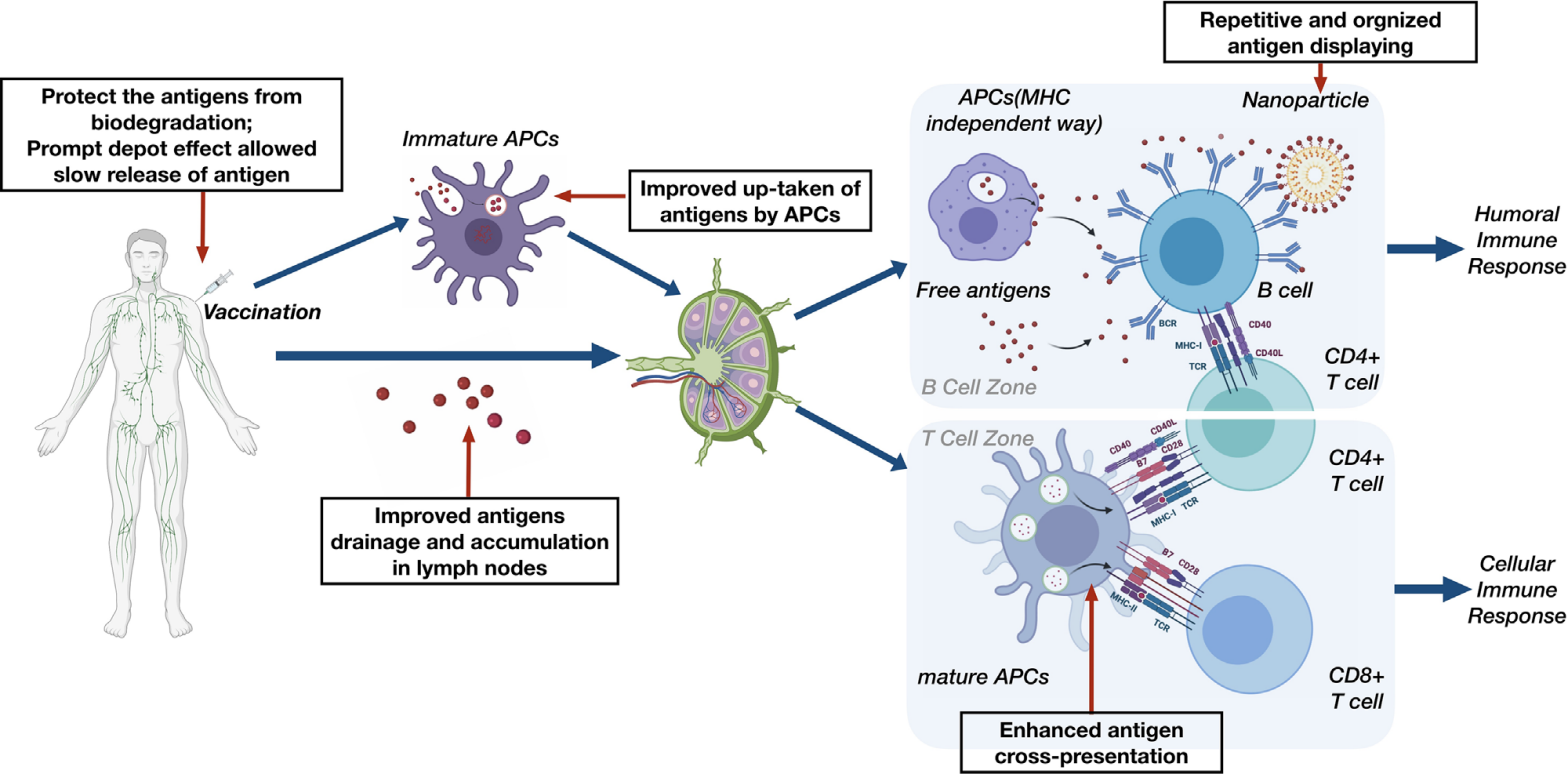
Schematic diagram of vaccination‐induced immune responses and the advantaged nanotechnology hold for improved vaccine development, which were placed in the black frames with thin red arrows. The cytokines interplay is not shown here. Created in BioRender.com (https://biorender.com/)

The general advantages of nanoparticles (NPs) for enhanced vaccine development are summarized in Figure 3.

## 3 NANOTECHNOLOGY ENABLED COVID‐19 VACCINE DEVELOPMENT

### 3.1 Lipid nanoparticles enable the development of mRNA‐based COVID‐19 vaccine

**FIGURE 4:**
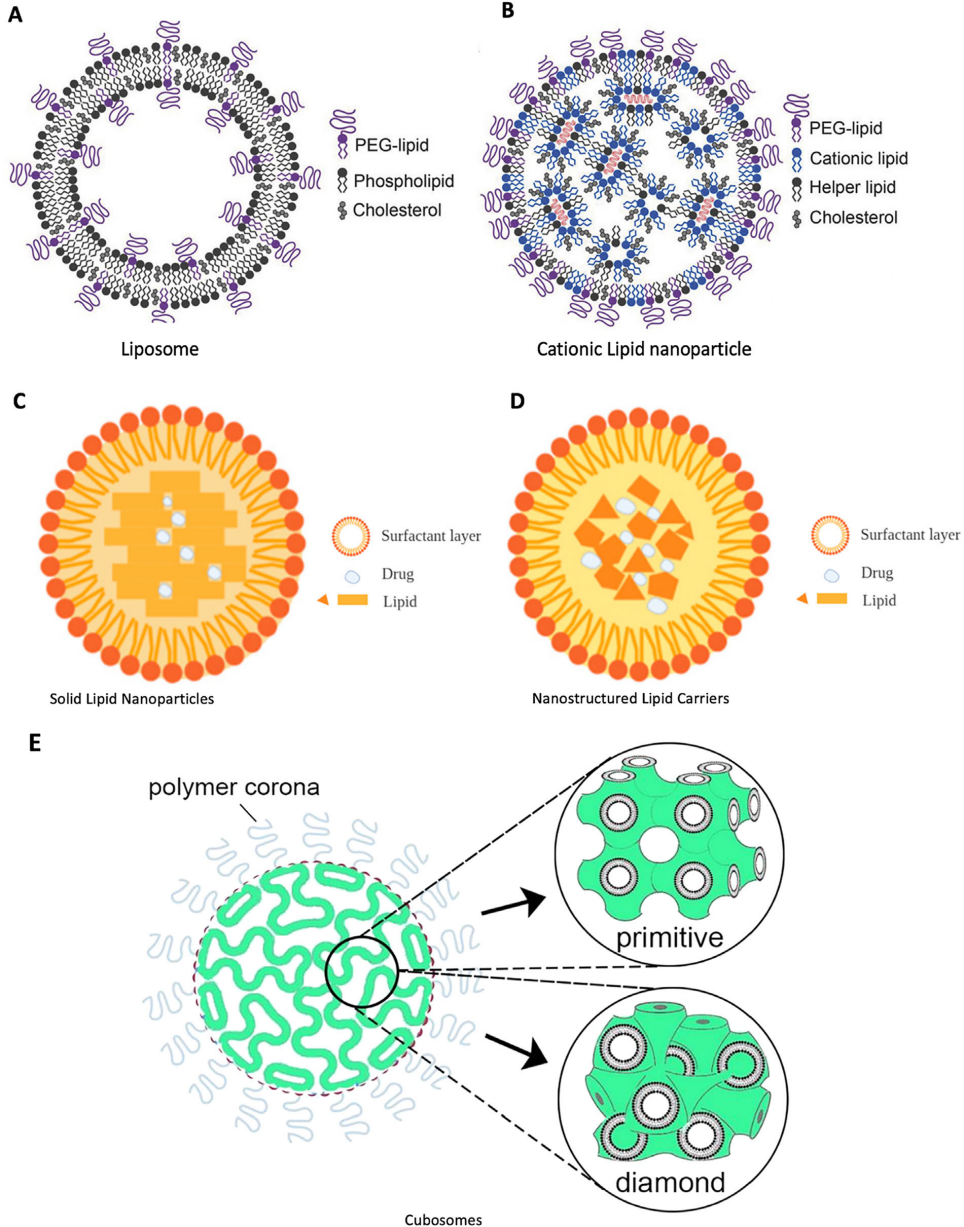
Schematic diagrams of lipid‐based nanoparticles. (A) Liposome and (B) cationic lipid nanoparticles.^[^ ^77^ ^]^ (A) and (B) are reproduced with permission.^[^ ^77^ ^]^ Copyright 2018, John Wiley and Sons. (C) Solid lipid nanoparticles (SLNs) and (D) Nanostructured lipid carriers (NLCs, imperfect type) have similar compositions, which include a surfactant layer and a solid lipid core that can encapsulate drugs, especially the lipophilic ones. The main limitation of SLNs is the low drug loading efficiency due to the perfect crystalline structure and possibility of drug expulsion due to the crystallization process under the storage conditions.^[^ ^62^, ^63^ ^]^ Nanostructured lipid carriers were designed to overcome this limitation by introducing imperfection into the structure and generally including an imperfect type, formless type, and multiple type.^[^ ^62^, ^63^ ^]^ (C) and (D) are reproduced under the terms of CC BY‐NC 4.0 license.^[^ ^62^ ^]^ Copyright 2020, MDPI. (E) Cubosome is one of the commonly used nonlamellar lipid nanoparticles, which are liquid crystalline particles with a highly stable structure, formed from lipid bio continuous bilayer cubic phases (either primitive or diamond type) and stabilized by polymer‐based outer coronas. Reproduced with permission.^[^ ^58^ ^]^ Copyright 2021, American Chemical Society

Lipid nanoparticles (LNPs) simultaneously play a key role in the development of COVID‐19 mRNA vaccines as vaccine carriers and adjuvants. The term “lipid nanoparticle” came into use in 1990s and evolved from “liposomes,” which were first described as early as the 1960s.^[^ ^53^, ^54^ ^]^ Liposomes are self‐assembled, sphere‐shaped vesicles comprising one or more concentric phospholipid bilayer(s) and an aqueous inner core (Figure 4A).^[^ ^55^, ^56^ ^]^ They are self‐assembled from phospholipids or synthetic amphiphiles with the assistance of sterols such as cholesterol, which can improve the stability of the liposomes by promoting the tight packaging of phospholipids and synthetic amphiphiles.^[^ ^57^ ^]^ They are typically of a size between 20 and 1000 nm, and from this perspective, they could be considered as the first generation of LNPs.^[^ ^58^ ^]^ Liposomes were considered potential drug carriers not long after being defined and turned out to be the first drug delivery platform that proceeded to clinical application with extraordinary versatility.^[^ ^59^ ^]^ This was attributed to their ability to transport both hydrophilic and hydrophobic drugs by incorporating the hydrophilic in the inner core, hydrophobic in the phospholipid bilayers, and good compatibility and biodegradability, which results in an excellent safety profile. In 1993, Crucell Berna Biotech from Switzerland designed and produced the first liposome‐based vaccine, Expaxal, a hepatitis A vaccine.^[^ ^60^ ^]^ This is composed of a formalin‐deactivated RG‐SB strain of hepatitis‐A virus that is incorporated into the phospholipid bilayer membrane of virosomes, which are special forms of liposomes that have virus envelope proteins incorporated into the lipid layers to facilitate the fusion of virosomes and endosome membranes.^[^ ^60^ ^]^

Based on the concept of liposomes, different types of LNPs have been developed to meet the need for drug delivery, including cationic LNPs (Figure 4B), solid LNPs (SLNs) (Figure 4C),^[^ ^61^, ^62^ ^]^ nanostructured lipid carriers (NLCs) (Figure 4D),^[^ ^61^, ^62^ ^]^ nonlamellar LNPs (NLNs) (Figure 4E),^[^ ^58^, ^63^ ^]^ ethosomes,^[^ ^64^ ^]^ and echogenic liposomes.^[^ ^65^ ^]^

The first LNP‐based RNA therapeutic was approved in 2018,^[^ ^58^ ^]^ and the latest successful application of LNP in vaccine development is in COVID‐19 mRNA vaccines. It has been a long time since the first observation in a mouse model that exogenously delivered mRNA could translate into proteins.^[^ ^66^ ^]^ However, several intrinsic features of nuclear acids hinder the development of nuclear acid‐based vaccines, including the negative charges and hydrophilicity of mRNA that lead to the difficulty of passive diffusion through plasma membranes, which are also negatively charged; moreover, RNA is highly susceptible to biodegradation.^[^ ^39^ ^]^ On the other hand, mRNA has several advantages as a vaccine antigen compared to whole‐virus, subunit proteins, and DNA. First, mRNA has a better safety profile because it is non‐infectious and has no potential risk of integration into the human genome, which may lead to tumorigenesis.^[^ ^67^, ^68^, ^69^, ^70^ ^]^ RNA is also easily biodegraded, and its half‐life can be controlled by chemical modification, leading to a controllable safety profile. Additionally, the development of mRNA vaccines is less time‐ and economy‐consuming compared to whole‐virus vaccines because it does not require the culturing of virus, and the in vitro transcription reactions are highly yielding.^[^ ^39^ ^]^ Lastly, ssRNA and double‐stranded RNA can be detected by the innate immune system as danger signals through TLR7/8 and TLR3, respectively,^[^ ^24^ ^]^ thus conferring intrinsic adjuvanticity to mRNA when used as vaccine components. This adjuvanticity could be further improved through modifications.^[^ ^71^ ^]^

An effective delivery platform is the key to realizing the concept of mRNA vaccines. The initial attempt using neutral liposomes to deliver oligonucleotides was impeded, owing to the low payload.^[^ ^39^ ^]^ Cationic LNPs, which were initially designed for drug delivery in cancer immunotherapy, displayed much higher encapsulation efficiency through charge interactions with anionic nucleic acids.^[^ ^72^ ^]^ Now, it is the most widely used nucleic acid delivery platform.^[^ ^73^ ^]^ mRNA‐1273^[^ ^74^ ^]^ and BNT162b2,^[^ ^75^ ^]^ two COVID‐19 vaccines parallelly designed by Moderna and Pfizer/BioNTech, respectively, are frontrunners of COVID‐19 vaccines, which both choose cationic LNPs to deliver the mRNA encoding the full‐length SARS‐CoV‐2 S protein. The mRNA is genetically engineered to express S proteins in a prefusion conformation, which greatly improves immunogenicity, and modifications including the introduction of two proline (2‐P) substitutions (K986P and V987P mutations) and the deletion of a furin cleavage site are adopted for the stable expression of prefusion S proteins.^[^ ^5^, ^74^, ^75^ ^]^

The composition and general structure of the LNPs used in these two vaccines are similar and are both assembled from ionizable lipids, PEGylated lipids, helper lipids (phospholipid distearoylphosphatidylcholine, DSPC), and cholesterol (Figure 4B). Patented ionizable lipids, SM‐102 (Moderna) and ALC‐0315 (Pfizer), were used.^[^ ^58^ ^]^ The ionizable lipids are characterized by a cationic head group linked to the hydrophobic lipid tails, which can electrostatically interact with mRNA, further forming an electron‐dense core in the presence of helper lipids and cholesterol, which are used to improve the stability of the membrane. PEGylated lipids are used to form a layer with helper lipids and cholesterol, and then wrap the electron‐dense core inside. The ultimate outcome was the formation of LNPs, with mRNA containing electron‐dense cores, surrounded by a PEGylated lipid monolayer, with a size ranging from 80 to 100 nm.^[^ ^76^ ^]^ The introduction of PEGylation is achieved by covalently attaching the polyethylene glycol (PEG) to the lipids, which is a frequently adopted strategy to improve the transportation efficiency and colloidal stability of lipid‐based NPs by avoiding the adsorption of serum protein, named “stealth effect.”^[^ ^77^, ^78^ ^]^ The above process proceeded with an increase in solvent polarity in a low pH (pH 4.0) solution to ensure the positive charge of the ionizable lipids so that the RNA could be effectively condensed.^[^ ^79^ ^]^

The ionizable lipids utilized have an acid dissociation constant (pKa) smaller than 7.0, which means they are neutral under physiological conditions,^[^ ^80^ ^]^ allowing their transportation in interstitial and lymphatic fluids, and once they enter into the acidified endosome compartments (pH < 6.0), the lipids are converted to be positively charged, followed by lipid exchange and fusion with the negatively charged endosome membrane, leading to the release of the mRNA into the cytoplasm, where translation into antigen proteins occurs.^[^ ^81^ ^]^ This process is called “endosome escape” as mentioned previously. Cationic lipids activate endosomal proton pumps and may assist the release of nucleic acids into the cytoplasm.^[^ ^81^ ^]^

According to the WHO, a total of 18 mRNA‐based COVID‐19 vaccine candidates have proceeded to clinical trials to date, most of which are based on ionizable LNPs similar to the aforementioned structure,^[^ ^6^, ^40^, ^82^, ^83^, ^84^, ^85^ ^]^ and several proprietary ionizable lipids have been used, including SM‐102 by Moderna,^[^ ^58^ ^]^ ALC‐0315 by Pfizer,^[^ ^58^ ^]^ ATX‐100 by Arcturus Therapeutics,^[^ ^86^ ^]^ CL1 by Genevant,^[^ ^87^ ^]^ and another one by Acuitas, respectively.^[^ ^88^ ^]^

**FIGURE 5:**
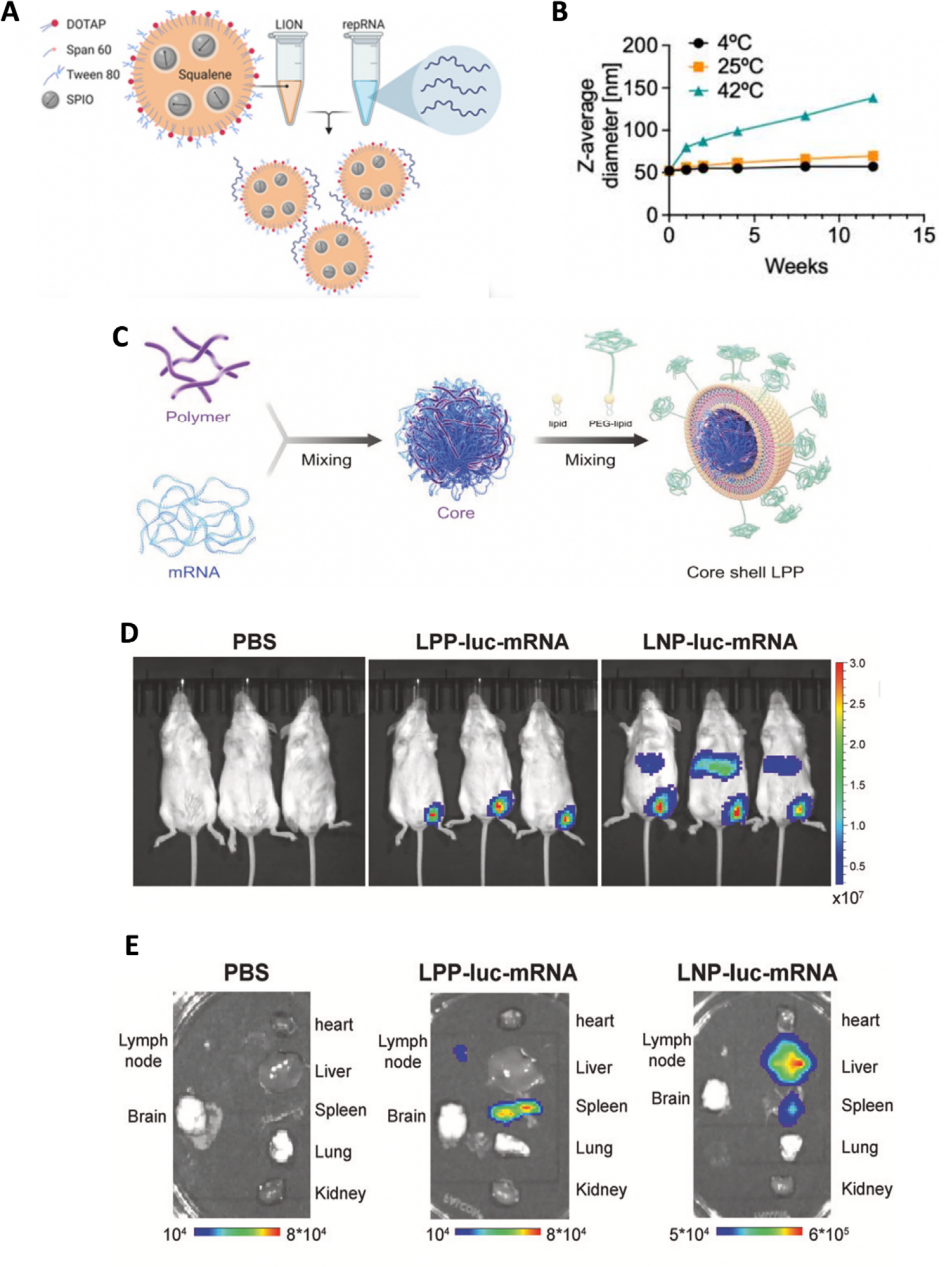
Other lipid‐based nanoparticles utilized in the mRNA vaccine candidate against SARS‐CoV‐2 that is different from cationic lipid nanoparticles (LNPs). (A) The structure of LION particles and the formation of the LION/repRNA‐CoV2S.^[^ ^89^ ^]^ (B) Change of the size of LION particles overtime during storage at 4°C, 25°C, or 42°C stable showed that LION particle can stay stable in 25°C for a long period.^[^ ^89^ ^]^ (A) and (B) are reproduced with permission.^[^ ^89^ ^]^ Copyright 2020, AAAS. (C) A graphical representation of the formation and composition of a core shelled lipopolyplex (LPP)‐based mRNA vaccine candidate (SW0123).^[^ ^91^ ^]^ (D) and (E) together show less accumulation of SW0123 in liver than that of the LNP‐based mRNA vaccines, which indicates a favorable biodistribution. (D) Biodistribution of LPP and LNP showed by bioluminescence in vivo.^[^ ^91^ ^]^ (E) Biodistribution of LPP and LNP showed by bioluminescence in isolated organs of mouse.^[^ ^91^ ^]^ (C–E) are reproduced under the terms of CC BY‐NC 4.0 license.^[^ ^91^ ^]^ Copyright 2021, Springer Nature

In addition to ionizable LNPs, other types of lipid‐based NPs have also been used. Erasmus et al. reported a self‐replicating mRNA vaccine encoding full‐length SARS‐CoV‐2 S protein, named LION/repRNA‐CoV2S, based on lipid inorganic NPs (LION).^[^ ^89^ ^]^ According to the authors, LION comprises a hydrophobic squalene core with inorganic superparamagnetic iron oxide (Fe_3_O_4_) NPs (SPIO)^[^ ^90^ ^]^ to improve the stability and span 60‐tween 80 on the surface to maintain the emulsion formation, as well as cationic lipid 1,2‐dioleoyl‐3‐trimethylammonium propane (DOTAP) for the complexation of the mRNA. The self‐replicating mRNA is incorporated into another vial named repRNA, which is a plasmid vector derived from Venezuelan equine encephalitis virus (VEEV). Lipid inorganic NPs and repRNA were produced separately, stored, and premixed before vaccination (Figure 5A). This new design led to great stability at room temperature for over 10 weeks (Figure 5B). Preclinical data showed that single dose of this candidate can induce a strong SARS‐CoV‐2 neutralizing antibody (NAbs) and type‐1 T helper cell response, with a modest S‐specific T cell response in nonhuman primates. This vaccine is now being further evaluated in a phase 1 clinical trial under the name HDT‐301 (NCT04844268).^[^ ^6^ ^]^

A similar strategy was adopted by the Infectious Disease Research Institute/Amyris, Inc., who took advantage of a well‐established RNA carrier used in ZIKV vaccine development,^[^ ^73^ ^]^ which has similar main components as LION, except for the replacement of SPIO with glyceryl trimyristate‐dynasan 114, a type of solid lipid. This candidate is about to proceed with a clinical trial.

Ren et al.^[^ ^91^ ^]^ reported an mRNA vaccine based on core shell‐structured lipopolyplex (LPP). The major difference between LPP and the LNP lies in the core of the particles that has a dense core formed by electrostatically complexing SW01 (a patented cationic polymer compound) and mRNA‐encoding full‐length S protein of SARS‐CoV‐2 (Figure 5C), which is different from the lipid‐mRNA mixed core of LNP. This resulted in a favorable biodistribution of antigens expressed, which may alleviate the systemic toxicity (Figure 5D,F), and this vaccine candidate can maintain stability at 4°C for up to 6 weeks. The application of liposome in COVID‐19 vaccines design is relatively limited, with only one candidate reported,^[^ ^92^ ^]^ which is composed of DOTAP and cholesterol. The vaccine can induce neutralizing and Th1‐biased immune responses via three different injection routes in mice, and it is now under evaluation in a phase 1 clinical trial (CTR20210542).^[^ ^6^ ^]^

All the mRNA‐based vaccines that currently enter the clinical trial phase were reported to induce NAbs immunity, along with Th‐1 skewed CD4+ response and CD8+ T cell immunity,^[^ ^5^ ^]^ which are significant improvements compared to traditional vaccines. This improvement can be attributed to the introduction of the LNPs. The “endosome escape” effect of cationic LNPs allows expression of antigens in the cytoplasm, followed by the digestion in proteasome and activation of MHC‐I mediated cross‐presentation pathway, resulting in CD8+ T cells activation along with costimulatory signals.^[^ ^40^ ^]^ Interestingly, Arcturus Therapeutics claimed that their mRNA vaccine candidate ARCO021 can protect the B cell‐deleted mice but not the CD8+ T cell‐depleted counterpart, indicating that the protection provided by the vaccine may be more T‐cell than B‐cell dependent, underlining the important role of T‐cell immunity in effective vaccination.^[^ ^93^ ^]^ In addition, previous studies on LNP‐based mRNA vaccines of the Zika virus (ZIKV), influenza, and human immunodeficiency virus (HIV) demonstrated that LNP administration induced a robust response of Tfh cells cooperating with GC B cells. This effect may also be achieved in the LNP‐based mRNA vaccine against Covid‐19 due to a similar strategy and may contribute to the observed strong humoral immune response.^[^ ^39^, ^44^, ^94^ ^]^

Most reported LNP‐based mRNA vaccines against SARS‐CoV‐2 do not contain additional adjuvants. The virus semblance of the LNPs was thought to contribute to this innate adjuvanticity,^[^ ^95^ ^]^ and mRNA can act as an adjuvant by stimulating DCs through TLR 3/7/8.^[^ ^96^ ^]^ Nucleoside substitution of uridine with pseudouridine or 1‐methylpseudouridine can further improve the adjuvanticity and stability of mRNA, and this strategy has been adopted by both Moderna^[^ ^97^ ^]^ and Pfizer/BioNtech.^[^ ^98^ ^]^ The avoidance of aluminum‐containing adjuvants decreases the possibility of a Th‐2 immune response for mRNA vaccines, which is associated with critical safety considerations, including eosinophil accumulation‐related severe lung damage.^[^ ^60^ ^]^ It is worth mentioning that PEG, which is widely used in the construction of LNPs, is possibly associated with IgE‐induced anaphylaxis and a Th‐2 skewing immune response, although this is still controversial.^[^ ^99^ ^]^ The amount of PEG was deliberately decreased to further improve the safety profile of some recently developed vaccines such as ARCT‐021.^[^ ^93^ ^]^

The composition and physicochemical properties also affect the adjuvanticity and performance of LNPs The unsaturated liposomes are more effectively taken up by macrophages than saturated liposomes.^[^ ^100^ ^]^ The size and surface charge also affect the uptake of LNPs by DCs.^[^ ^32^, ^101^, ^102^ ^]^ Lipid NPs with smaller sizes and positive charges are able to effectively accumulate in draining lymph nodes, leading to more potent activation of the immune system compared to larger ones. Moreover, LNPs can also be functionalized to increase the reactivity of targeting immune cells; for instance, the incorporation of immunodominant CTL epitope peptides within liposomes can induce a highly efficient antiviral CD8+ T‐cell response.^[^ ^103^ ^]^

### 3.2 Self‐assembled protein nanoparticles (SAPN): An access to better subunit/peptide‐based COVID‐19 vaccines

Self‐assembled protein NPs represent another versatile tool for COVID‐19 vaccine development. Their application in this field has significantly advanced the concept of subunit‐ and epitope‐based vaccines. Briefly, nanotechnology realized this through two strategies: to encapsulate or conjugate the subunit/peptide in SAPN as a delivery platform or to complex the subunits themselves into antigen NPs to improve immunogenicity.

**FIGURE 6:**
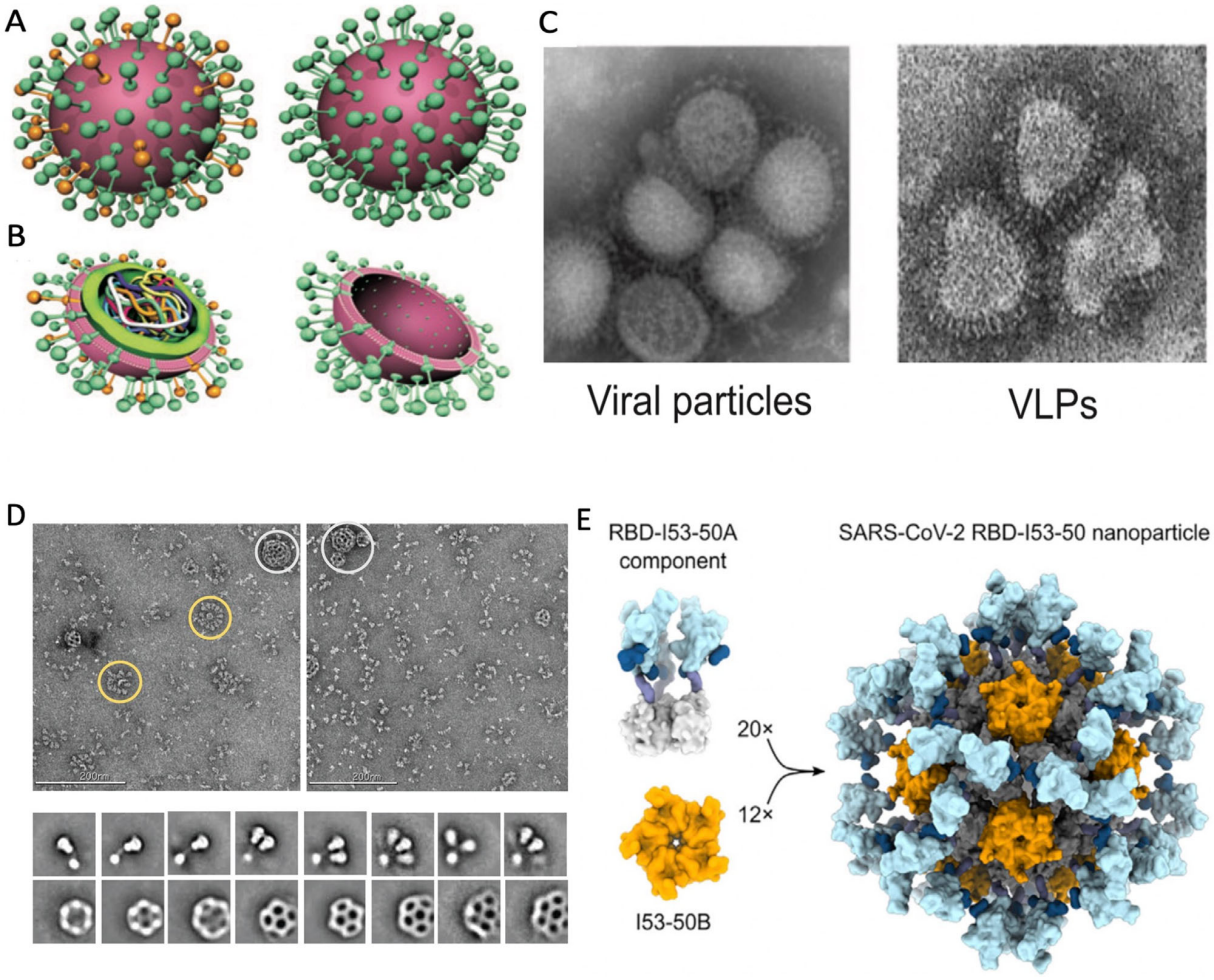
Self‐assembled protein nanoparticles (SAPN). (A–C) Schematic representation of viral particles, VLPs, and synthetic virus‐like particles (SVLPs). Reproduced with permission.^[^ ^104^ ^]^ Copyright 2010, John Wiley and Sons. (A) Comparison of external characteristics of virus (left) and VLPs (right).^[^ ^104^ ^]^ (B) Internal comparison of virus particle (left) and VLPs (right).^[^ ^104^ ^]^ (C) Comparison of influenza viral particles (left) and influenza VLPs (right) under transmission electron microscopy.^[^ ^104^ ^]^ (D) A highly structured subunit vaccine candidate assembled viral morphology. Each particle has an icosahedral 120 subunit core (153‐50B), conjugated to 60 SARS‐CoV‐2 RBD‐153‐50A components at the 153‐50B‐interactive domain. 153‐50A and 153‐50B are designed in silico.^[^ ^138^ ^]^ Reproduced under the terms of CC BY‐NC 4.0 license.^[^ ^138^ ^]^ Copyright 2020, Elsevier. (E,F) Vaccine candidate NVX‐CoV2373 from Novavax. Reproduced with permission.^[^ ^140^ ^]^ Copyright 2020, AAAS. (E) Multitrimer rosettes formed S protein subunits of SARS‐CoV‐2 indicated with yellow circles, and admixed with Matrix‐M indicated with white circles in PS 80. Images under negative stain electron microscopy.^[^ ^140^ ^]^ (F) Images of individual spikes, spike nanoparticles, and Matrix‐M clearly.^[^ ^140^ ^]^

In the first strategy, both viral and non‐viral proteins were utilized. Viral protein self‐assembled NPs resembling the natural structure of enveloped viruses are called VLPs (Figure 6A–C).^[^ ^104^ ^]^ They can be considered as “empty shells” of viruses without nucleic acid material, with a size of 15–30 nm.^[^ ^105^ ^]^ Various systems can be used to produce VLPs, including prokaryotic cells, yeasts, insects, plants, and mammalian cells.^[^ ^106^ ^]^

VLPs offer multiple advantages in terms of vaccine development. First, they provide a particulate formation that is associated with a significantly higher efficacy of uptake by APCs and accumulation in the draining lymph nodes compared with the free, soluble protein subunits.^[^ ^45^ ^]^ Additionally, VLPs enable repetitive and organized display of antigens on their surfaces. Another prominent advantage of VLPs as vaccine delivery platforms is their ability to activate CD8+ T cells via the cross‐presentation pathway.^[^ ^107^ ^]^ Cytotoxic CD8+ T cells are responsible for eliminating intracellular pathogens. As recently reported, a preponderant CD8+ T response is associated with mild COVID‐19 infection.^[^ ^108^ ^]^ This advantage has led to great interest in VLP‐based vaccines for the treatment of cancer and chronic diseases.^[^ ^109^, ^110^, ^111^, ^112^, ^113^ ^]^ The effective stimulation of a CD8+ T cell response and generation of CD8+ memory cells is now also deemed a vital perspective of vaccine development for infectious diseases.^[^ ^114^ ^]^ Though it is well‐established that antigens presented by VLPs are prone to cross‐presentation,^[^ ^115^ ^]^ the underlying mechanism remains to be fully elucidated. It seems to be proteasome^[^ ^116^, ^117^ ^]^ and transporter associated with antigen processing (TAP)‐independent,^[^ ^118^ ^]^ which apparently differs from the pH‐sensitive cross‐presentation induced by the endosome escape of cationic LNPs. It has been reported that the peptides displayed by VLPs are taken up in a clathrin‐dependent manner before being degraded in lysosomal compartments, and then form MHC‐I complexes with MHC‐I molecules recycled from the cell surface, thus achieving cross‐presentation activation.^[^ ^41^ ^]^ In addition, it is still controversial whether the cross‐presentation of VLP‐derived peptides is restricted to CD8+ DCs, given that contradictory observations exist.^[^ ^41^, ^119^ ^]^

Despite the dimness of the mechanism, VLPs are widely deployed in vaccine design. There are now six VLP‐based vaccine candidates against SARS‐CoV‐2 that have proceeded into the clinical trial phase, and 18 are under pre‐clinical evaluation according to the WHO.^[^ ^6^ ^]^ The frontrunner of VLP‐based vaccines includes Medicago Inc.’s recombinant coronavirus virus‐like particle (CoVLP) produced from *Nicotiana benthamiana*, a relative of the tobacco plant, which is highly susceptible to a special plant‐specific bacterium *Agrobacterium* infection. *Agrobacterium* containing the DNA sequence encoding the S protein of SARS‐CoV‐2 was infiltrated into the leaves of *N. benthamiana*, followed by harvest and purification of VLPs 4–6 d thereafter.^[^ ^120^ ^]^ This relatively simple procedure confers higher speed and better scalability compared to those of other platforms, which are the most predominant advantages according to the manufacturer, who claimed that this platform is capable of developing clinical‐grade vaccines within 6–8 weeks.^[^ ^120^ ^]^ In the interim report of a phase 2 clinical trial (NCT04636697),^[^ ^121^ ^]^ the NAbs level and Th response profile of CoVLP adjuvanted with AS03, a patented oil‐in‐water emulation adjuvant by GSK,^[^ ^27^ ^]^ were assessed in adults and older adults. Two doses of vaccination astoundingly induced a ∼10‐fold higher level of NAb compared to that in convalescent patients (27–105 d after onset of symptoms), and ELISpot revealed both Th‐1 and Th‐2 responses by measuring the number of interferon (IFN)‐γ and IL‐4 expressing T cells, respectively. The robust humoral immunity in vaccinated populations may be attributed to an unexpected Th‐2 response, which is related to Tfh cell involvement. No severe adverse events were reported, except for the Th‐2 response; however, the safety profile will be carefully assessed in the ongoing phase 2/3 clinical trial (NCT04636697).^[^ ^6^ ^]^ Another candidate currently studied in a phase 2 clinical trial is an alum‐adsorbed vaccine expressing HexaPro‐S, M, N, and E proteins of either wild SARS‐CoV‐2 or B.1.1.7 variant strain and adjuvanted with CpG ODN (NCT04962893).^[^ ^6^ ^]^ HexaPro‐S is a newly reported prefusion S protein variant with six proline substitutions (S‐6P) that leads to a significant improvement in protein yield and stability under heat stress as well as freeze‐thaw cycling.^[^ ^122^ ^]^ This candidate is the only one‐dose vaccine among the six VLP‐based candidates that proceeded into clinical trials and was administered subcutaneously (Table 1).

**TABLE 1:** Virus‐like particles in clinical trials or clinical use

**VLP in clinical trials**			
**Candidate vaccine**	**Description of the vaccine**	**Developers**	**Status**
RBD SARS‐CoV‐2 HBsAg VLP Vaccine	A subunit vaccine where the RBD antigen is conjugated to the hepatitis B surface antigen to allow the stimulation of the immune system	Serum Institute of India, Accelagen Pty, SpyBiotech	Phase 1/2
CoVLP COVID‐19 Vaccine	Using CoVLP technology, which consists of recombinant spike‐in(S) glycoproteins expressed as VLP, administered with GSK's plant‐derived adjuvant, it is the only plant‐based neo‐crown vaccine in the world	Medicago Inc.	Phase 3
VBI‐2902a, VBI‐2905a	An eVLP of SARS‐CoV‐2 spike (S) glycoprotein and aluminum phosphate adjuvant	VBI Vaccines Inc.	Phase 1/2
SARS‐CoV‐2 VLP Vaccine	An alum adsorbed vaccine expressing HexaPro‐S, M, N, E proteins of either wild SARS‐CoV‐2 or B.1.1.7 variant strain, and adjuvanted by CpG ODN	The Scientific and Technological Research Council of Turkey	Phase 2
ABNCoV2	Using the cVLP technology +/− adjuvant MF59	Radboud University	Phase 1
LYB001	A RBD from SARS‐CoV‐2 and VLP vector, adjuvanted with aluminum hydroxide	Yantai Patronus Biotech Co., Ltd.	Phase 1
UB‐612	Consists of 8 components (S1‐RBD‐sFc fusion protein, 6 synthetic peptides (1 universal peptide and 5 SARS‐CoV‐2‐derived peptides), an adapted CpG TLR‐9 agonist, and aluminium phosphate adjuvant) that induce potent neutralizing antibodies and broad‐spectrum T‐cell responses against SARS‐CoV‐2	United Biomedical	Phase 2/3
EpiVacCorona	Contain three chemically synthesized peptide antigens of the SARS‐CoV‐2 S protein, and the epitopes that may cause ADE of infection are avoided deliberately	Federal Budgetary Research Institution State Research Center of Virology and Biotechnology “Vector”	Phase 3
CoVac‐1	A peptide‐based vaccine candidate, composed of SARS‐CoV‐2 T cell epitopes derived from various viral proteins, combined with the Toll‐like receptor 1/2 agonist XS15 emulsified in Montanide ISA51 VG, aiming to induce profound SARS‐CoV‐2 T cell immunity to combat COVID‐19	University Hospital Tübingen	Phase 2

VLP in preclinical trials^[^ ^3^ ^]^		
Description of the vaccine	Target antigen	Developers
VLP	SARS‐CoV‐2	Max Planck Institute for Dynamics of Complex Technical Systems
VLP‐based DC‐targeting vaccine	SARS‐CoV‐2	University of Manitoba
VLP	SARS‐CoV‐2	Bezmialem Vakif University
Enveloped eVLP	SARS‐CoV‐2, SARS‐CoV, & MERS‐CoV	VBI Vaccines Inc.
S protein integrated in HIV VLPs	SARS‐CoV‐2	IrsiCaixa AIDS Research/IRTA‐CReSA/Barcelona Supercomputing Centre/Grifols
VLP + Adjuvant	SARS‐CoV‐2	Mahidol University/ The Government Pharmaceutical Organization (GPO)/Siriraj Hospital
VLP, lentivirus, and baculovirus vehicles	SARS‐CoV‐2	Navarrabiomed, Oncoimmunology group
VLP, based on RBD displayed on virus‐like particles	SARS‐CoV‐2	Saiba GmbH
ADDomerTM multiepitope display	SARS‐CoV‐2	Imophoron Ltd and Bristol University's Max Planck Centre
Unknown	SARS‐CoV‐2	Doherty Institute
VLP	SARS‐CoV1, SARS‐CoV‐2	OSIVAX
eVLP	SARS‐CoV‐2	ARTES Biotechnology
VLPs peptides/whole virus	SARS‐CoV‐2	University of Sao Paulo
VLPs produced in BEVS	SARS‐CoV‐2	Tampere University
Plant‐derived VLP	SARS‐CoV‐2	Shiraz University
Myxoma virus co‐expressing S, M, N, and E proteins	SARS‐CoV‐2	Arizona State University
Plasmid driven production of VLPs containing S, M, N, and E proteins of SARS‐CoV‐2	SARS‐CoV‐2	Arizona State University
VLP with RCB	SARS‐CoV‐2	Berna Biotech Pharma

Abbreviations: BEVS, baculovirus expression vector system; CoVLP, coronavirus‐like particles; cVLP, capsid virus‐like particle; DC, dendritic cell; eVLP, enveloped virus‐like particle; RBD, receptor‐binding domain; VLP, virus‐like particles.

Heterogeneous viral proteins were utilized to develop VLPs as a COVID‐19 vaccine carrier, including those from HBV,^[^ ^106^ ^]^ influenza,^[^ ^123^ ^]^ myxoma virus,^[^ ^6^ ^]^ lentivirus, and baculovirus.^[^ ^6^ ^]^ Antigens are either genetically or chemically conjugated to the surface of VLPs. Frontrunners of this kind are vaccine candidates by Accelagen Pty, which is composed of SARS‐CoV‐2 RBD proteins displayed on the surface of HBsAg‐assembled VLPs (RBD SARS‐CoV‐2 HBsAg VLP vaccine, details not stated), which is under evaluation in a phase 1/2 clinical trial in Canada (ACTRN12620000817943).^[^ ^6^ ^]^

Besides generating VLPs, non‐viral protein‐based NPs or proteinaceous biomaterial scaffolds have also been widely investigated as vaccine carriers, including ferritin,^[^ ^124^, ^125^ ^]^ vault protein,^[^ ^126^, ^127^ ^]^ and encapsuling, etc.^[^ ^128^ ^]^ Among these, ferritin has been the most extensively studied. Ferritin is a ubiquitously existing iron‐containing protein, self‐assembled into 24 subunits, and formed as a spherical cage‐like NP with a size of ∼12 nm.^[^ ^129^ ^]^ Each ferritin NP has eight threefold axes on its surface, with readily accessible solvent, which confers ferritin the potential to be an antigen‐displaying platform, with hemagglutinin (HA) of influenza virus being the first case of this strategy.^[^ ^130^ ^]^ The HA is genetically conjugated to ferritin. The first step was the construction of the HA‐ferritin fusion gene by linking the ectodomain of HA with ferritin. The final product was ferritin NPs with HA antigens displayed on the surface with a size of ∼20 nm. HA nanoparticles could yield a 7.2‐fold higher HA inhibition than that of an inactivated influenza vaccine containing the same antigens in the presence of an adjuvant.^[^ ^130^ ^]^

The same strategy has been applied to COVID‐19 vaccine development. The SARS‐CoV‐2 spike ferritin nanoparticle (SpFN) is a COVID‐19 vaccine candidate produced per the abovementioned protocol by fusing the ectodomain of the prefusion S protein of SARS‐CoV‐2 with ferritin.^[^ ^131^ ^]^ The SARS‐CoV‐2 spike ferritin nanoparticle paired with Alhydrogel^®^ (ALFQ), a liposomal formulation and alum‐containing adjuvant, could provide broad neutralizing protection in nonhuman primates.^[^ ^132^ ^]^ The SpFN+ ALFQ‐induced T‐cell response spectrum was further assessed in mice. The results showed that vaccination with SpFN+ALFQ could induce robust APC and T cell responses, with Th‐1 skewing immunity. Above all, K ^b^ spike (539‐546)‐memory CD8+ T cells with effective cytolytic functions were found in the lungs of vaccinated mice.^[^ ^131^ ^]^ Based on these favorable preclinical data, SpFN+ALFQ was used in a phase I clinical trial (NCT04784767).^[^ ^6^ ^]^ Several other candidates based on similar strategies are under preclinical evaluation.^[^ ^6^, ^133^, ^134^, ^135^ ^]^ without using genetic conjugation, Wang et al. utilized an in‐house developed SpyTag/SpyCatcher technique‐based click vaccine platform^[^ ^136^ ^]^ to form an antigen‐ferritin nanoparticle vaccine against SARS‐CoV‐2 by linking Spy‐tagged RBD to ferritin‐SpyCatcher.^[^ ^137^ ^]^ Another representative case was reported by Walls et al.,^[^ ^138^ ^]^ who constructed an icosahedral subunit core comprised of 120 in silico‐designed components; the icosahedral subunit core was able to display 60 SARS‐CoV‐2 S‐RBD proteins on the surface in a highly immunogenic array (Figure 6D), which was able to elicit NAbs titers 10‐fold higher than that in the free prefusion‐stabilized spike antigen, with a five‐fold lower dose.^[^ ^138^ ^]^

Vaccines using subunits self‐assembled into NPs are another form of nanotechnology‐facilitated vaccine development. A total of 37 subunit vaccine candidates proceeded to clinical trials.^[^ ^6^ ^]^ ZF001, produced by Anhui Zhifei Longcom Biopharmaceutical and Institute of Microbiology, Chinese Academy of Sciences, is the first subunit vaccine that received Emergency Use Authorization, EUA. The ZF001 immunogen is comprised of two RBDs expressed in the Chinese hamster ovary (CHO) cell system, which are then fused together via a disulfide‐link or tandem repeat.^[^ ^139^ ^]^

The main purpose of assembling the subunits into NPs is to improve the immunogenicity of antigens through particulate formation as well as clustered and repetitive antigen display. The structures of subunit NPs are more flexible and diverse than those of VLPs. NVX‐CoV2373 by Novavax represents one of the current frontrunners of this kind, which utilizes the self‐assembly of stabilized, full‐length 2‐P S subunits of SARS‐CoV‐2 reconstructed in polysorbate 80 (PS 80). The subunits self‐cluster into structures ranging from free trimers to as many as 14 trimer transmembrane domains incorporated in micellar PS 80 cores displaying multitrimer rosettes (Figure 6E,F).^[^ ^140^ ^]^ The vaccine contains a proprietary NP adjuvant matrix M, which is a mixture of 40‐nm‐sized honeycomb‐like NPs derived from purified *Quillaja saponaria* Molina saponin with cholesterol and phospholipids.^[^ ^141^ ^]^ Co‐formulation with Matrix‐M was reported to yield significantly increased humoral and cellular immune responses compared to that with non‐adjuvanted antigens.^[^ ^142^ ^]^ Data from a phase 3 clinical trial showed 90.4% overall efficacy against symptomatic COVID‐19 infections, 93.2% against eight viral variants of interest (VOI) and concern (VOC), and 100% protection against moderate and severe diseases.^[^ ^143^ ^]^

Protein‐based vaccines are usually stored for a longer period than mRNA vaccines. In addition, they have the potential to form complex subunit combinations and tertiary structures to improve their performance. However, compared to the in vivo expression of mRNA vaccines, the heterologous expression of protein‐based vaccines may lead to a lack of post‐translational modifications of the natural antigen, including glycosylation, which is especially important for COVID‐19 vaccine design, given that the SARS‐CoV‐2 S protein has up to 22 N‐linked and several O‐linked glycosylation sites.^[^ ^86^ ^]^ In line with this, the S‐RBD protein of SARS‐CoV‐2 contains two N‐linked glycans (N331, N343) and two O‐linked glycans (T323 and S325).^[^ ^86^ ^]^ The glycosylation of antigens may lead to various immunogenetic effects in different scenarios. Glycosylation may increase the ability of antigen particles to target germinal centers of lymph nodes by activating the complement system.^[^ ^144^ ^]^ In addition, a higher glycosylation rate may improve the interaction of antigens with APCs by interacting with lectins (glycan‐binding proteins) on the surface of APCs, and glycosylation at certain molecular sites is important for antigen stabilization. Glycosylation may impact the vaccine‐induced immune response by shielding immunogenic epitopes.^[^ ^86^ ^]^ Thus, defining the optimal S or S‐RBD glycoforms of SARS‐CoV‐2 for vaccine‐induced immune responses and antigen stabilization will be important for the production of an optimized protein‐based vaccine.

An epitope‐based vaccine is another promising subunit vaccine. It utilizes only the immunogenic part of the antigens and is superior to other subunit vaccines in terms of low cost and high specificity.^[^ ^5^ ^]^ Emerging epitope‐mapping tools and bioinformatics approaches capable of epitope prediction greatly accelerate the process of epitope‐based vaccine development.^[^ ^145^, ^146^, ^147^ ^]^ Frontrunners of this kind against SARS‐CoV‐2 include UB‐612, EpiVacCorona, and CoVac‐1.^[^ ^6^ ^]^ UB‐612 is the first multitope protein/peptide vaccine combining different epitopes. It contains an S1‐RBD‐sFc fusion protein for B cell epitopes, one universal peptide, and five synthetic Th/CTL peptides derived from SARS‐CoV2 S2, M, and N proteins for class MHC‐I and II molecules. High titers of NAbs and a strong TH1 skewing and cellular immune response were observed in vaccinated animals, and a phase 2/3 clinical trial is ongoing (NCT04683224).^[^ ^148^ ^]^ EpiVacCorona contains three chemically synthesized peptide antigens of the SARS‐CoV‐2 S protein, and epitopes that may cause antibody‐dependent enhancement (ADE) of infection are deliberately avoided. The peptides were conjugated to a protein carrier and adsorbed onto an aluminum‐containing adjuvant.^[^ ^149^ ^]^ EpiVacCorona is currently under evaluation in a Phase 3 clinical trial (NCT04780035).^[^ ^6^ ^]^ Epitope‐based vaccines have a great potential for future applications. Through multiple epitope combinations, different purposes may be achieved, such as improving the cooperation of T and B cell immune responses^[^ ^150^ ^]^ or development of a pan‐coronavirus vaccine through selection of relatively conserved or cross‐reactive coronavirus regions.

### 3.3 Cell‐membrane‐derived nanoparticles for vaccination

Cell‐membrane‐based nanocarriers retain the natural composition and function of the original cell membrane, a property that is difficult to achieve with any other platform.^[^ ^151^, ^152^ ^]^ While other types of nanocarriers can also be artificially modified to exhibit different surface compositions, it is difficult to express the complex proteins on the surface of cell membrane‐derived carriers that have better biocompatibility and the ability to induce an efficient immune response. Thus, cell membrane‐derived NPs offer a platform with great potential for therapeutic and vaccine development applications in tumors or infectious diseases.

Cell membrane‐derived nanoscale vesicles can be derived from engineered cell membranes or actively secreted and released by different cell types such as tumor cells, red blood cells, or cells of bacterial origin. Exosomes are extracellular vesicles (EVs) secreted by eukaryotic cells.^[^ ^153^ ^]^ In recent years, they have been explored as highly stable drug delivery system with good targeting capabilities, owing to their inherent tissue homing ability. Natural substance components with different functions on the surface of EVs contribute to better targeting, intracellular penetration, and controlled release of drugs.^[^ ^154^ ^]^ In addition, EVs can be designed to present viral antigens and thus induce highly specific T and B cell responses, highlighting the great potential for application in vaccine development. Compared to other delivery vectors such as liposomal NPs, which are also based on phospholipids or viral vectors, key features based on EVs^[^ ^155^, ^156^ ^]^ include (1) preservation of the original antigenic conformation, (2) lower immunogenicity, (3) less toxicity, and (4) ability to cross biological barriers, provide additional advantages for their safe and effective application in vaccine development.

Engineered EVs are cell membrane‐derived nanocarriers that can be designed and optimized for different targets and functional requirements. A study based on an engineered EV platform reported^[^ ^157^ ^]^ that by designing a chimeric S protein as a transmembrane structure that replaced the G protein on the surface of exosomes secreted by vesicular stomatitis virus (VSV), the S protein load could be significantly increased. Recently, several biotechnology companies have been developing EV‐based vaccines against COVID‐19 by delivering mRNAs that express the SARS‐CoV‐2 structural protein via EVs. For example, Capricor Therapeutics has developed two different SARS‐CoV‐2 vaccines using an EV‐based platform:^[^ ^158^ ^]^ one using vectors of the four structural proteins S, N, M, and E of SARS‐CoV‐2 transfected into HEK293 cells, releasing EVs (or VLPs) that carry the full range of viral antigens in their original conformation. This nano‐vector vaccine carrying multiple proteins may induce a more effective immune response in an organism.^[^ ^159^ ^]^ Alternatively, COVID‐19 vaccines have been formulated by loading mRNAs of full‐length S proteins and modifying the four structural proteins of SARS‐CoV‐2 inserted into Lamp1 proteins in EVs for better presentation of MHC I and II molecules. Recently, several studies have reported that exosomal vaccines loaded with mRNA elicit durable cellular and humoral responses and produce fewer adverse effects compared to those of COVID‐19 vaccines currently in clinical use or in development. Ciloa's two‐component CoVEVax vaccine consists of an EV carrying primers that allow the production of spike DNA (DNAS‐EV) and a booster, which allows it to induce an effective humoral and cellular immune response in mice without any adjuvant, exerting a neutralizing response.^[^ ^158^ ^]^ Versatope Therapeutics designed a bacterial outer membrane vesicle (OMV) that displays the RBD of spike protein by fusing it to the OMV‐anchoring protein cytolysin A (ClyA).^[^ ^159^ ^]^ Polak et al. recently reported that an EV‐based vaccine encloses the mouse viral envelope protein and induces neutralizing antibodies (NAbs) and cellular immune responses, thus eliminating the need for adjuvants.^[^ ^158^ ^]^

Nanodecoy is another kind of cell membrane‐derived nanoparticle, and by fusing genetically engineered cell membrane‐derived nanovesicles stably expressing virus surface antigen receptor, the nanodecoy can effectively adsorb viruses as its name ‘decoy’ indicated, thus having a potential role in the treatment of virus infection, which has been well described by others.^[^ ^161^, ^162^, ^163^, ^164^ ^]^ On the other hand, given the ability to naturally trap pathogens, the resulted nanodecoy‐pathogen complexes could also be applied to vaccine development. Typically, we use thermal or chemical methods to eliminate or reduce the infectivity of the virus for vaccine preparation, however, this process may lead to the alterations in virus antigens and reduction in immunogenicity, nanodecoy‐virus complex may provide a facile way to develop safe and effective vaccines by preserving structural integrity and immunogenicity of the virus.^[^ ^151^ ^]^

In conclusion, cell membrane‐derived NPs provide a simple route for the development of safe and effective vaccines. It has several properties that facilitate the development of vaccines. First, the small size facilitates antigen presentation through blood circulation; second, pathogens or toxins can be displayed on the surface of membrane‐derived NPs and can be further modified to retain their immunogenicity better and adapt them to different applications; and finally, membrane‐derived NPs can be taken up by target cells through endocytosis, facilitating the localization and metabolism of pathogens. Together, these properties contribute to the safety and efficacy of the vaccines.

### 3.4 Nanotechnology enabled DNA vaccine delivery

DNA vaccines are another type of vaccine that has made rapid progress against SARS‐CoV‐2. The premier platform for the delivery of DNA vaccines is an adenovirus vector, which falls out of the scope of this review and has been comprehensively reviewed elsewhere.^[^ ^165^ ^]^ The frontrunners of this kind of nanotechnology‐driven product include Ad5nCoV designed by CanSino, ChAdOx1‐S (AZD1222) by Oxford/AstraZeneca, and Ad26.COV2.S by Janssen Pharmaceutical, all of which were approved for clinical application.^[^ ^6^, ^165^ ^]^ Very recently, ZyCoV‐D, a needle‐free plasmid DNA vaccine produced by Zydus Cadila, received EUA from the Drug Controller General of India (DCGI) based on interim results from a phase 3 trial that showed 66.6% and 100% efficacy in preventing symptomatic and moderate disease against the *Delta* variant, respectively,^[^ ^166^ ^]^ thus becoming the first plasmid DNA vaccine approved for clinical use.

Another elegant case of nanotechnology for DNA vaccine delivery is the candidate produced by Entos Pharmaceuticals Inc., which utilizes a patented proteolipid vesicle (PLV) for this purpose. PLVs are based on Entos’ Fusogenix Platform, which is formulated with neutral lipid layers incorporated within a proprietary fusion‐associated small transmembrane protein,^[^ ^167^ ^]^ a unique membrane fusion catalyst encoded by fusogenic reoviruses that can induce syncytium formation and promote lipid exchange and fusion of membranes. Based on this, Entos PLVs can effectively fuse with the plasma membrane of target cells and deposit the loaded vaccine directly into the cytoplasm by bypassing the endocytic pathway, thus improving the efficiency of vaccine entry into the cytoplasm. Entos claims that this platform is universal for the delivery of a range of cargos, including DNA, mRNA, miRNA, *CRISPR*, etc.^[^ ^167^ ^]^ The vaccine is now evaluated in a phase I clinical trial (NCT04591184).^[^ ^6^ ^]^ Additionally, electroporation has been implemented for some DNA vaccine candidates to improve the efficacy of plasmid DNA entering host cells by temporarily improving membrane permeability. An example of this strategy is INO‐4800 from Inovio Pharmaceuticals, which is injected intradermally with electroporation by a proprietary device Cellectra^[^ ^168^ ^]^ and is now evaluated in a phase 2/3 clinical trial (NCT04642638).^[^ ^6^ ^]^

In contrast to mRNA vaccines, DNA plasmids are first translocated into the nucleus for transcription before antigen expression in the cytoplasm, whereas mRNA vaccines can be directly translated into the cytoplasm after escape from endosomal compartments, thus being able to produce more antigens at smaller doses. The major advantage of DNA vaccines is their stability, which leads to long‐lasting expression compared with mRNA.^[^ ^5^ ^]^

DNA vaccines have several merits, including lower cost, lower requirements of storage conditions owing to their stability, and readiness for quick development of new vaccines for emerging variants.^[^ ^169^ ^]^ The main difficulty with DNA vaccines is how to effectively pass through the plasma membrane and translocate into the nucleus. Nanotechnology may play a role in solving this problem because of its ability to efficiently deliver cargo and target cellular components. It is worth noting that, although rare, DNA vaccines carry the potential risk of host genome integration.^[^ ^170^ ^]^ This needs to be considered when developing a DNA‐based vaccine.

## 4 CONCLUSION AND PERSPECTIVE

Nanomaterial holds advantages including ultra‐small and controlled‐size particle dimensions and the ability to have multi‐functionalization possibilities (surface modification, targeted drug delivery, biocompatibility, slow and sustained drug release) endow them with great potential in the field of disease treatment and prevention.^[^ ^171^, ^172^ ^]^ However, the challenges still exist for the application of different types of nanoparticle carriers. Lipid NPs are favored by vaccine developers because they have a high drug delivery efficiency and are easily manufactured. The current use of microfluidics to generate LNPs, which encapsulate mRNA in LNPs before entering the body, is the only FDA‐approved mRNA delivery technology on the market, and its safety and efficacy have been demonstrated in this pandemic.^[^ ^173^ ^]^ However, improving the stability of LNP vaccines remains a challenge, particularly in the case of non‐thermal formulations. Although materials such as polyethylene glycol can improve the stability of the system, recent reports have shown that mRNA cargoes in Pfizer/Biotech and Moderna's COVID‐19 vaccine must still be held at ultra‐low temperatures to maintain their stability.^[^ ^174^ ^]^ To address this issue, the application of engineered nanoscale transport systems can help to improve the overall thermal stability of the cargo carried, and optimizing the appropriate route of administration can help to improve the effectiveness and durability of drug‐ or vaccine‐induced immune protection.

Protein assembly based vaccine candidates are another powerful strategy due to their plasticity and diversity.^[^ ^175^ ^]^ The use of protein assemblies has several advantages over other nanocarriers:^[^ ^175^, ^176^, ^177^, ^178^ ^]^ (1) they allow multiple pathogenic units to be retained in an assembled structure, maintaining the original activity of the assembly; (2) repetitive surface patterns and a particulate structure that triggers a strong immune response, and (3) they can be designed to prevent enrichment and amplification of pathogenic components. However, for generic vaccine applications, the selection of VLP candidates based on the type of immune response and the development of effective design strategies remain challenging. As some optimization measures, screening for better linkers may help present target antigens more efficiently on the surface of APCs, while natural building blocks, structural biology, and computation may also provide additional guidance for the rapid design and development of protein assembly candidates.^[^ ^175^ ^]^

Cell membrane‐derived NPs provide an attractive platform for therapeutic and vaccine development applications against infections. Compared to other vaccines, cell membrane‐based vectors such as exosomes have lower immune activity and higher uptake rates than those of LNPs or adenovirus, reducing the need for booster doses.^[^ ^154^ ^]^ However, the industrial production of cell membrane‐derived NPs is difficult, and their characterization of the immune response induced by different diseases needs to be further investigated.

In general, the long‐accumulated interdisciplinary experience of nanotechnology and vaccine immunology hugely facilitated the development of vaccines. Challenges still exist for the development of an ideal vaccine in the future, including but not limited to lower lost, lower requirements of storage conditions, improved safety profile, and enhanced cooperation between different immune components. Though we still have a long way to go, the introduction of nanotechnology has placed more options for the development of an ideal vaccine against COVID‐19.

## CONFLICT OF INTEREST

The authors declare no conflict of interest.
